# Imaging Active Drug Transport in Viable Human Skin: A Functional ex vivo Skin Model for Mechanistic and Regulatory Applications

**DOI:** 10.1159/spp/aetag009

**Published:** 2026-09-25

**Authors:** Krishna Chaitanya Telaprolu, Jonathan Wei, Jeffrey E. Grice, Yousuf Mohammed, Michael S. Roberts

**Affiliations:** a Therapeutics Research Centre, Frazer Institute, The University of Queensland, Woolloongabba, QLD, Australia; b Department of Biomechanical Engineering, Faculty of Mechanical, Maritime and Materials Engineering, Delft University of Technology, Delft, The Netherlands; c School of Pharmacy, The University of Queensland, Woolloongabba, QLD, Australia; d UniSA—Clinical and Health Sciences, University of South Australia, Adelaide, SA, Australia; e Therapeutics Research Centre, Basil Hetzel Institute for Translational Medical Research, The Queen Elizabeth Hospital, Woodville, SA, Australia; f Therapeutics Research Centre, Centre for Clinical Research, The University of Queensland, Woolloongabba, QLD, Australia

**Keywords:** Viable epidermis, P-glycoprotein, multiphoton microscopy, drug diffusion, topical delivery, Pharmacokinetics

## Abstract

**Introduction:**

Understanding drug diffusion and active transport in the viable epidermis (VE) is critical for optimizing topical and transdermal drug delivery. However, the VE remains understudied due to challenges in isolating physiologically intact membranes and limited functional data on cutaneous transporters. This study aimed to develop and validate a viable human epidermal model to assess molecular diffusion and transporter activity using high-resolution imaging, with a focus on P-glycoprotein (P-gp)-mediated transport.

**Methods:**

Viable epidermis was enzymatically separated from dermatomed human skin using dispase II. Tissue viability was confirmed by MPT–FLIM, MTT assay, and H&E staining. Multiphoton microscopy (MPM) was employed to visualize the diffusion of rhodamine 123 (RD123), a fluorescent P-gp substrate, in dispase-separated epidermis (DSE), and heat-separated epidermis (HSE). The influence of P-gp was evaluated by pretreatment with verapamil, a known inhibitor. ImageJ and MATLAB were used for quantitative image analysis.

**Results:**

RD123 diffusion was faster and more extensive in verapamil-treated DSE, with increased fluorescence intensity in the basal and suprabasal layers at early time points compared to controls. HSE exhibited significantly reduced fluorescence and compromised cellular architecture. MPT–FLIM analysis confirmed that the average NAD(P)H fluorescence lifetime and a_1_/a_2_ metabolic ratios in DSE were comparable to control DTM, indicating preservation of metabolic activity.

**Conclusion:**

This study establishes a viable, structurally intact epidermal model suitable for evaluating both passive and active transport in human skin. Functional imaging confirmed the role of P-gp in modulating drug diffusion across the VE. This platform supports the generation of mechanistic data for improving dermal pharmacokinetic models and has translational relevance for formulation design and regulatory assessment of topical therapies.

## Introduction

Human skin is a complex and heterogeneous barrier composed of morphologically distinct layers that play critical roles in regulating molecular transport. Drug penetration into and through the skin depends on various factors, including the physicochemical properties of the molecule, formulation characteristics, and local skin environment [1]. A quantitative understanding of drug distribution across skin layers is essential for optimizing topical and transdermal delivery systems for both therapeutic and cosmeceutical applications [2].

Most skin pharmacokinetic models segment the skin into the stratum corneum (SC), viable epidermis (VE), and dermis, based on structural and functional distinctions [3]. Experimental approaches for evaluating drug distribution across these layers can be broadly categorized into invasive, minimally invasive, and non-invasive methodologies [4]. Minimally invasive approaches, including tape stripping and microdialysis [5, 6] enable partial depth profiling of drug penetration in vivo, although they are constrained by limited spatial resolution and potential perturbation of the skin barrier. Non-invasive techniques, such as confocal and multiphoton microscopy, confocal Raman spectroscopy, optical coherence tomography, quantitative autoradiography, and mass spectrometry imaging, allow spatially resolved and, in some cases, real-time assessment of drug distribution while preserving tissue architecture [7, 8]. A detailed review of non-invasive methods for skin penetration was discussed by Darvin et al. in his review [4]. While these techniques provide complementary insights, each has inherent limitations in terms of spatial resolution, quantitative capacity, and tissue preservation [2, 9–11].

The SC is widely recognized as the principal barrier to drug penetration [12]. However, the viable epidermis also contributes to drug disposition, yet permeability parameters for this layer are less well characterized, largely due to challenges in isolating viable, structurally intact epidermis [13, 14]. Ellison et al. recently estimated viable epidermal partition coefficients for 50 compounds using indirect measurements comparing isolated and whole epidermis [15, 16]. Nevertheless, there remains a paucity of direct data on solute diffusion within the viable layers of human skin. Such data are critical for developing and validating mechanistic skin models [12], which can facilitate formulation design and regulatory assessment.

Imaging methods have emerged as powerful tools to visualize drug diffusion in skin. Confocal microscopy has been used to monitor fluorescent molecule distribution in unfixed skin [2], while fluorescence recovery after photobleaching (FRAP), applied via multiphoton microscopy, has enabled quantification of diffusion coefficients in the SC and viable epidermis [17, 18]. Additional studies have used confocal and multiphoton imaging to assess macromolecular diffusion following minimally invasive delivery [19, 20]. The viable epidermis, however, imposes additional barriers, limiting the diffusion of both small and large molecules, including sulforhodamine, albumin, insulin, and inactivated vaccines [21]. Tight junctions further restrict paracellular transport of small hydrophilic compounds [22, 23]. In this context, multiphoton microscopy offers a compelling balance, enabling high-resolution, depth-resolved imaging in intact skin with reduced photodamage and improved penetration compared to conventional confocal techniques [24]. This makes it particularly well suited for resolving drug distribution within viable epidermal sublayers. This positioning is consistent with recent reviews highlighting the growing role of advanced optical imaging techniques in bridging the gap between invasive quantification and non-invasive visualization of dermal drug delivery [4].

Despite this, most modeling efforts still focus on the SC, with limited data on the distinct sublayers of the viable epidermis, namely, the stratum granulosum (SG), stratum spinosum (SS), and stratum basale (SB). Improved understanding of solute distribution across these sublayers is necessary for accurate prediction of skin pharmacokinetics.

In addition to passive diffusion, active transport processes may play a significant role in the disposition of topically applied drugs. Skin expresses several ATP-binding cassette (ABC) and solute carrier transporters involved in xenobiotic handling [25–28]. P-glycoprotein (P-gp; ABCB1), for example, has been implicated in the dermal absorption of corticosteroids using knockout models [29], while organic anion transporting polypeptides (OATPs) have been functionally characterized in keratinocytes [30]. Osman-Ponchet et al. demonstrated P-gp and MRP1 activity in human skin using rhodamine 123 (RD123) and verapamil as a specific inhibitor [31], and Li et al. further established the role of MRP1 in efflux transport using knockout mice [32]. These findings suggest that modulation of transporter activity may offer a strategy to enhance drug localization in the skin while minimizing systemic exposure [29, 33].

Despite emerging evidence, the functional role of transporters in human skin remains underexplored, particularly under physiological conditions. Quantifying drug diffusion and transporter function at the cellular level in viable skin would provide important mechanistic insights and improve the predictive power of dermal pharmacokinetic models.

To address this, we developed a viable epidermal membrane model by enzymatic separation using dispase II. The structural integrity and metabolic viability of the membrane were confirmed via MPT–FLIM imaging and MTT assays. We selected rhodamine 123 (RD123), a fluorescent substrate of P-gp [31, 34, 35], and used multiphoton microscopy to assess its diffusion across viable epidermal sublayers. Verapamil, a known P-gp inhibitor [36], was used to evaluate transporter mediated modulation of RD123 distribution.

Quantitative image analysis was performed using ImageJ and MATLAB. Comparison of RD123 diffusion in inhibitor-treated versus control membranes enabled assessment of transporter function in viable epidermis. This approach, combining a physiologically relevant skin model with high-resolution imaging, provides a novel platform to study active transport and passive diffusion in human skin, with implications for improved dermal drug delivery strategies.

## Materials and Methods

### Human Skin Samples

The skin was donated following elective surgery procedure, with ethical approval granted by the Metro South Princess Alexandra Hospital and The University of Queensland Human Research Ethics Committee (HREC/16/QPAH/064). After collecting, the skin was cleaned by removing subcutaneous fat using a surgical scalpel blade, and the cleaned skin was used immediately.

### Chemicals

Dispase II (CatLog no. D4693; enzymatic activity: 0.70 U/mg), Thiazolyl Blue Tetrazolium Bromide, HEPES (4-(2-Hydroxyethyl) piperazine-1-ethanesulfonic acid, Phosphate buffered saline sachets (pH 7.4), Sodium chloride, Dimethyl sulfoxide, Rhodamine 123 (RD123), and agarose were purchased from Sigma Aldrich Australia. The medium Dulbecco’s modified Eagle’s medium (1X), Fetal bovine serum and Anti-biotic-Antimycotic (100X) were purchased from Thermo Fisher Scientific Australia. All solutions were prepared using Milli-Q water.

### Preparation of Epidermal Membranes

Human skin membranes were prepared from freshly excised skin and provided with informed consent by donors undergoing elective abdominoplasty. All three donor skins used in these studies were from female individuals aged between 45 and 55 years. The acquisition and use of the donated skin were approved by the Metro South Hospitals and the University of Queensland Human Research Ethics Committees (Approval number: 2008001342). The subcutaneous fat was removed, and the skin was dermatomed at 500 µm thickness using an Integra^®^ Padgett^®^ air dermatome (Model C Padgett instruments, INC., Miami Princeton, FL, USA). Epidermal membranes were prepared by heat separation by dipping the skin in water at 60°C for 2 min and scraping the epidermis from dermis using blunt end of spatula. The separation of epidermis by dispase II enzyme was developed by using dermatomed skin of 500 µm thickness. The finalized procedure for separation of epidermis is the use of split-thickness skin (500 µm) of chosen length (8 × 5 cm) was placed on sterile gauze in a tray containing 7 mL of the prepared Dispase II solution (2.4 IU/mL in HEPES buffer pH 7.4) and incubated at 37°C in a water bath for 90 min. After incubation, the skin was removed from the gauze, and the epidermis was scraped off with a metal spatula and washed with triple distilled water for 30 s to remove any traces of enzyme before being used for evaluation of epidermal viability and for other ex vivo experimental purposes.

### Viability Testing

#### Viability Testing by MPT-FLIM

The endogenous autofluorescence of the separated epidermis was imaged using the multi photon tomography DermaInspect^®^ (JenLab GmbH, Jena, Germany). The imaging system consists of Plan-Neofluar high-NA oil-immersion 40X/1.30 objective lens (Carl Zeiss, Germany). The light for excitation was provided by the ultra-shortpulsed, mode-locked, 80-MHz, Titanium Sapphire laser (Mai Tai, Spectra Physics, Mountain View, CA, USA) with a tuning range of 710–920 nm and an 85-fs pulse width. The endogenous cellular autofluorescence of NAD(P)H was imaged by using an excitation wavelength of 740 nm (two photon), at an optical power of 23 mW and at an exposure of 47 s with an acquisition image size of 512 × 512 pixels (256 × 256 µm^2^). A bandpass filter of 350–650 nm (BG39, Schott glass color filter) was used to optically filter the emitted fluorescence light to capture live images of MPT or toward the Fluorescence lifetime imaging microscope (FLIM) detectors. In front of FLIM detectors, 350–450 nm (channel 1) and 450–515 nm (channel 2) band pass filters were used to isolate NAD(P)H and FAD signals, respectively, into separate channels. FLIM measurements were carried out by using a time-correlated single-photon counting SPC-830 detector system (Becker & Hickl, Berlin, Germany) integrated to the MPT system. Leite-Silva et al. described the FLIM system in detail used for the study. We acquired images of the stratum granulosum layer and stratum basale, at a specific depth determined by keratinocyte morphology [37, 38].

#### Data Analysis of MPT Images for Viability

The FLIM data were analysed using SPCImage software version 5.3 (Becker & Hickl GmbH, Berlin, Germany) data analysis package [39]. The images were processed using the double-exponential fit with mean lifetime (τ_m_) as a decay parameter. After the analysis, the distribution histograms of pixel intensity versus the average lifetime (τ_m_) were exported. The pixel frequency data were used to normalise the intensity to any given area. The same images were also processed using the decay parameter which is a ratio (a_1_/a_2_) of amplitude percentage (a_1_%) of fast decaying component (τ_1_) correspond to free NAD(P)H divided by the amplitude percentage (a_2_%) of slow decaying component (τ_2_) corresponds to bound NAD(P)H. The histograms of pixel intensity versus a_1_/a_2_ ratio was also exported. The images of the stratum granulosum and stratum basale of all the membranes (control DTM skin, dispase separated epidermis (DSE), and heat separated epidermis (HSE)) were pseudo coloured to qualitatively display the mean lifetime weighted by their relative contributions of free and pro-tein-bound NAD(P)H [40].

#### Viability Testing by MTT Assay

Separated epidermal membranes, dermis remained after enzymatic epidermis separation and control DTM skin were cut into discs of 2.4 cm^2^ area and placed in 6 well plate with 1 mL DMEM media containing 1% anti-anti and 1% fetal bovine serum (FBS). The well plate was kept in an incubator at 37°C with 5% CO_2_ overnight (18 ± 2 h). After overnight incubation, MTT assay was carried out by adding 1 mL of MTT to each well with the membrane (0.3 mg/mL in PBS) and incubating for 90 min at 37°C with 5% CO_2_. After incubation, the excess MTT was removed, and membranes were washed with PBS. The formed formazan was extracted with 1 mL of DMSO and the absorbance measured at 575 nm using Thermo Scientific™ Multiskan™ FC Microplate photometer.

#### Hematoxylin and Eosin (H&E) Staining

The epidermis separated by Dispase enzyme and heat was fixed along with the control DTM skin as a positive control using 10% formalin. The fixed tissues were embedded in paraffin and sectioned with 4 µm thickness and stained with H&E solutions. The images were captured using Olympus Slide scanner VS120 (rm4026) optical microscope and viewed/assessed by using Olyvia software.

### Preparation of Agarose Solution and RD123 Solution

Agarose gel (0.1%) was prepared in PBS by heating 0.05 g of agarose in 50 mL of PBS. After complete dissolution of agarose, the solution was kept under magnetic stirring until it reached room temperature to form a uniform gel. RD123 (5 µm) in 0.1% agarose gel was prepared by adding 25 µL of 1 mm RD123 solution to 5 mL of agarose gel and vortexing for 1 min to form a uniform solution.

### Inhibitor Treatment

RD123 used in the study is a substrate for the P-glycoprotein (P-gp) [41], for which verapamil was used as an inhibitor (P-glycoprotein/MDR1 inhibitor) [36, 42]. An epidermal membrane of 19 mm diameter was incubated in DMEM media spiked with the inhibitor to achieve a final inhibitor concentration of 100 µm. The incubation was carried out for 2 h at 37°C followed by washing the membrane with PBS for 3–4 times to remove any surface adsorbed inhibitor. The washed membrane was used for imaging.

### Multiphoton Fluorescence Microscopy Imaging of RD123 Diffusion

The optimised procedure involves placing the epidermis on the coverslip with the stratum basale side exposed to the air interface. The edges of the skin were covered with wet gauze to prevent drying of the epidermis while imaging (Fig. 1a). RD123 (5 µm) incorporated in 0.1% agarose gel prepared in PBS was used as donor solution for imaging. A LaVision Biotec Multiphoton microscope consisting of a Nikon Ti-U inverted microscope (Bielefeld, Germany) equipped with a 60 × 1.2 numerical aperture (NA) water immersion lens (Olympus, Tokyo, Japan) and a Titanium: Sapphire femtosecond-pulse laser (Spectra Physics Mai-Tai, Santa Clara CA, USA) was used for imaging of the epidermis (Fig. 1b). A stack of 500 × 500 μm images with a pixel resolution of 512 × 512, along with the Z-plane with a step size of 2 μm to a total depth of 60 µm was imaged at every scan. The first stack of images was obtained using an excitation wavelength of 760 nm with power set to 7% to allow the morphology of the skin layers to be clearly seen. A 200 µL donor solution was added in the cylindrical donor chamber placed on the top of the skin. To image RD123, an excitation wavelength of 1,010 nm (two photon) was used at 40% laser power. Each replicate was first imaged for 3 min in fast 3D time-lapse scan mode to capture the initial sub minute kinetics. The imaging was then repeated every 5 min for 30 min to capture the equilibration stage of molecular diffusion occurring in the layers and every 10 min for 30 min thereafter to capture any depletion that may occur. A total of 10 stacks was collected, requiring a total imaging time of approximately 60 min. The emitted light was separated into three channels using three bandpass filters (460–510 nm; 535–585 nm; and 605–675 nm), with one raw image stack generated per emission channel. The filter setup is shown in Figure 1b. Multiphoton TIFF format images were processed using ImageJ 1.51v (National Institutes of Health, USA). RD123 was detected in the second emission channel (λex: 535–585 nm) and images from this channel were used for the analysis. By using the above-mentioned optimised conditions, DSE and HSE membranes of the same donor were used to determine the diffusion process of the molecules. The role of active transport was studied by comparing the diffusion process of the molecules in the live membrane (DSE), live membrane treated with transport inhibitors and inactivated membrane (HSE).

**Fig. 1.**
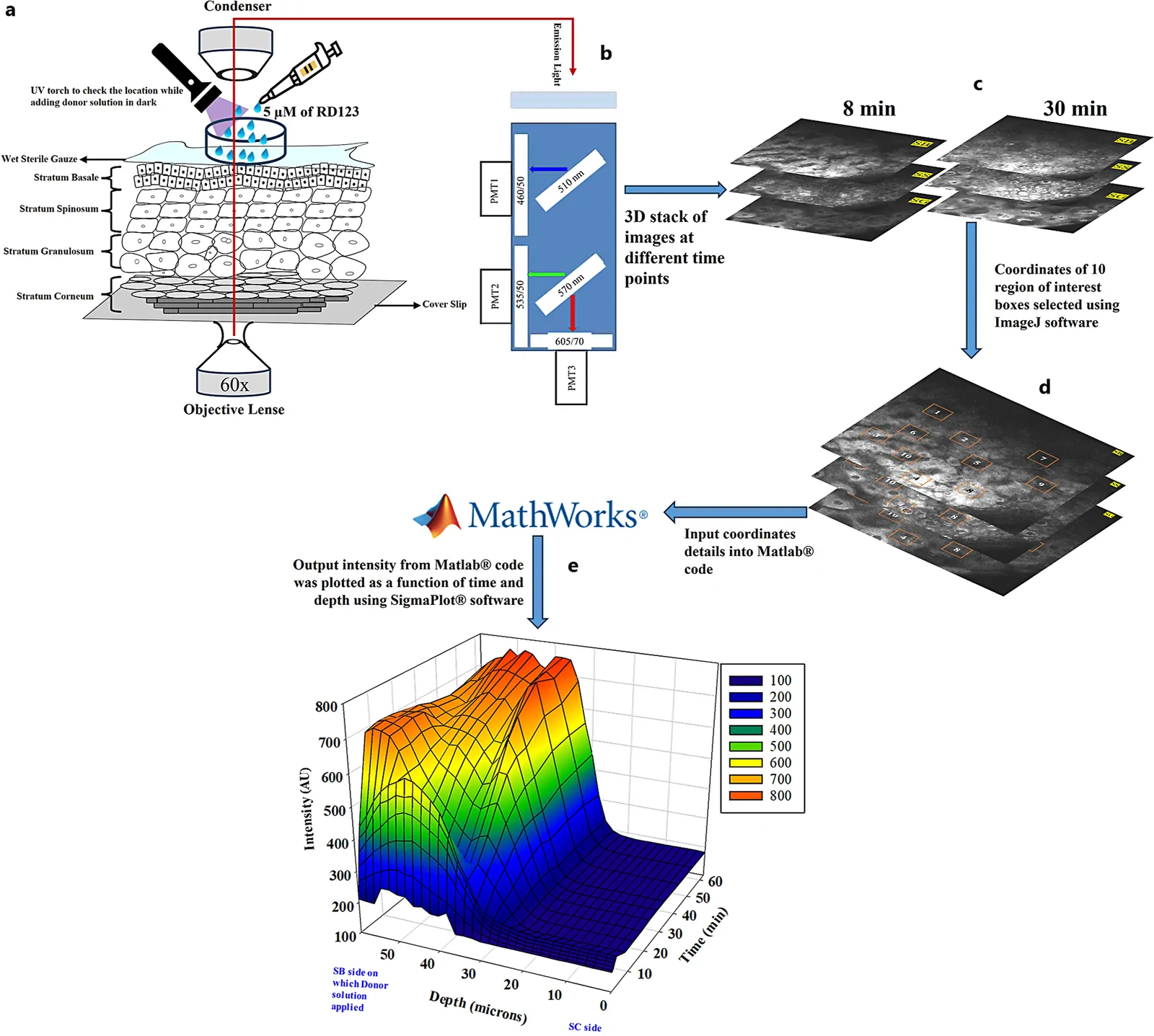
**a** Microscopy imaging set up of epidermis with stratum basale toward air interface and the edges covered by wet tissue and cylindrical donor chamber placed on top epidermis to confine the area of imaging and prevent the spreading of the donor solution. **b** Filter setup showing the arrangement of bandpass filter to cut-off the emitted light into different channels. **c**, **d** Representative SB, SS, and SG skin layer from a stack at 8 min and 30 min was shown and the ROI boxes were drawn at different areas of the imaged skin. **e** Coordinates of these boxes were given as input into Matlab code to get the output as intensity in each layer which is plotted as intensity versus depth versus time in a 3D mesh plot.

### Diffusion Analysis – Mean Intensity in a Boxed Region over the Entire Z-STACK

Images from the second emission channel (λex: 535–585 nm) in a 16-bit TIFF format were used to measure the fluorescent intensity of the RD123 in SG, SS and SB layers of human skin. In the imaging area, ten 50 × 50 (length × width) region of interest (ROI) pixel boxes was drawn using ImageJ at rete pegs i.e., a ridge of the epidermis at the zone of separation in the separated epidermis and non-rete regions, to capture the representative diffusion process happening in the skin as shown in Figure 1c and d. These coordinates were used as input in Matlab code (Matlab R2016b/R2017a Math Works, MA, USA) (Supplementary file) and the average intensity of all ten boxes in each layer at each time point are produced as the Matlab output. These data were used to plot intensity-depth-time plots in a 3D mesh using SigmaPlot 13.0 version (Systat Software Inc.) as shown in Figure 1e. To improve the understanding of the data, average intensity of three donors was plotted as intensity versus time only at specified depth for both control and inhibitor treated skin.

### Statistical Analysis

Mean fluorescence intensity values of RD123 in control and inhibitor-treated viable epidermis were compared using a two-way analysis of variance (ANOVA), with treatment and epidermal layer as factors, followed by Šídak’s multiple comparisons test (GraphPad Prism v9, GraphPad Software, San Diego, CA, USA). Data are from *n* = 3 donors and are presented as mean ± SEM. A *p* value <0.05 was considered statistically significant.

## Results

### Viability Testing

#### Viability Testing by MPT-FLIM

The MPT-FLIM is used to visualize morphological structures of various layers of skin by using endogenous fluorophores autofluorescence and any changes that occur in the native state can be seen by noninvasive by MPT-FLIM. Figure 2a and b shows the representative pseudo-coloured autofluorescence images of stratum granulosum of the DSE and control DTM skin, respectively, with an average lifetime (τm) range between 0 and 2,000 ps from the same skin donor. Figure 2c and d shows representative pseudo-coloured autofluorescence images of stratum basale of the DSE and control DTM skin, respectively, with an average lifetime (τm) range between 1,000 and 3,500 ps from the same skin donor. In HSE (Fig. 2e) the specific layers of the membrane could not be identified. The change in NAD(P)H lifetime determined by FLIM analysis was used to differentiate the necrosis and apoptosis of the cells [43]. The average lifetime (τ_m_) of both control DTM skin and DSE was ∼1,200 ps in 3 different donors (Table 1), indicating no significant changes in autofluorescence of DSE after separation compared to control DTM skin. The average lifetime of NAD(P)H is ∼ 1,000 and any change in average lifetime of NAD(P)H is a sign of progressive ischaemic necrosis in the treated skin compared to control [40]. The average lifetime (τ_m_) of HSE was ∼250 ps, corresponding most likely to the keratin component of the cellular walls and any signs of cellular autofluorescence of NAD(P)H was absent in HSE. The average lifetime τ_m_ is a sensitive indicator of cellular health and metabolic status and changes in the microenvironment of the fluorophore are detectable by a deviation from the unaffected lifetime [40]. All skin types are made up of differing proportions of the endogenous fluorophores and hence the lifetime of the control skin from the same donor (in this instance the control DTM skin) is generally regarded as a benchmark. Figure 2f shows the histograms of the pixel intensity versus NAD(P)H average lifetime (τm). Figure 2g shows pixel intensity versus the ratio (a1/a2) of amplitude percentage (a1%) of the fast-decaying component (τ1) corresponding to free NAD(P)H, divided by the amplitude percentage (a2%) of the slow decaying component (τ2), corresponding to bound NAD(P)H. In Figure 2f, major peaks at ∼1,300 ps and ∼1,200 ps were observed in DSE and control DTM skin, respectively, while a peak at ∼250 ps was observed in HSE. In Figure 2g, a major peak at an a1/a2 ratio of ∼2 was observed in both DSE and control DTM skin. The a1/a2 ratio is indicative of changes in the metabolic state of the cells and also used to understand the physiology of the cells [44]. We observed no change in the a1/a2 ratio of DSE compared to control DTM skin (Table 1), indicating that the bio physiology of the separated epidermis is similar to that of the control DTM skin.

**Fig. 2.**
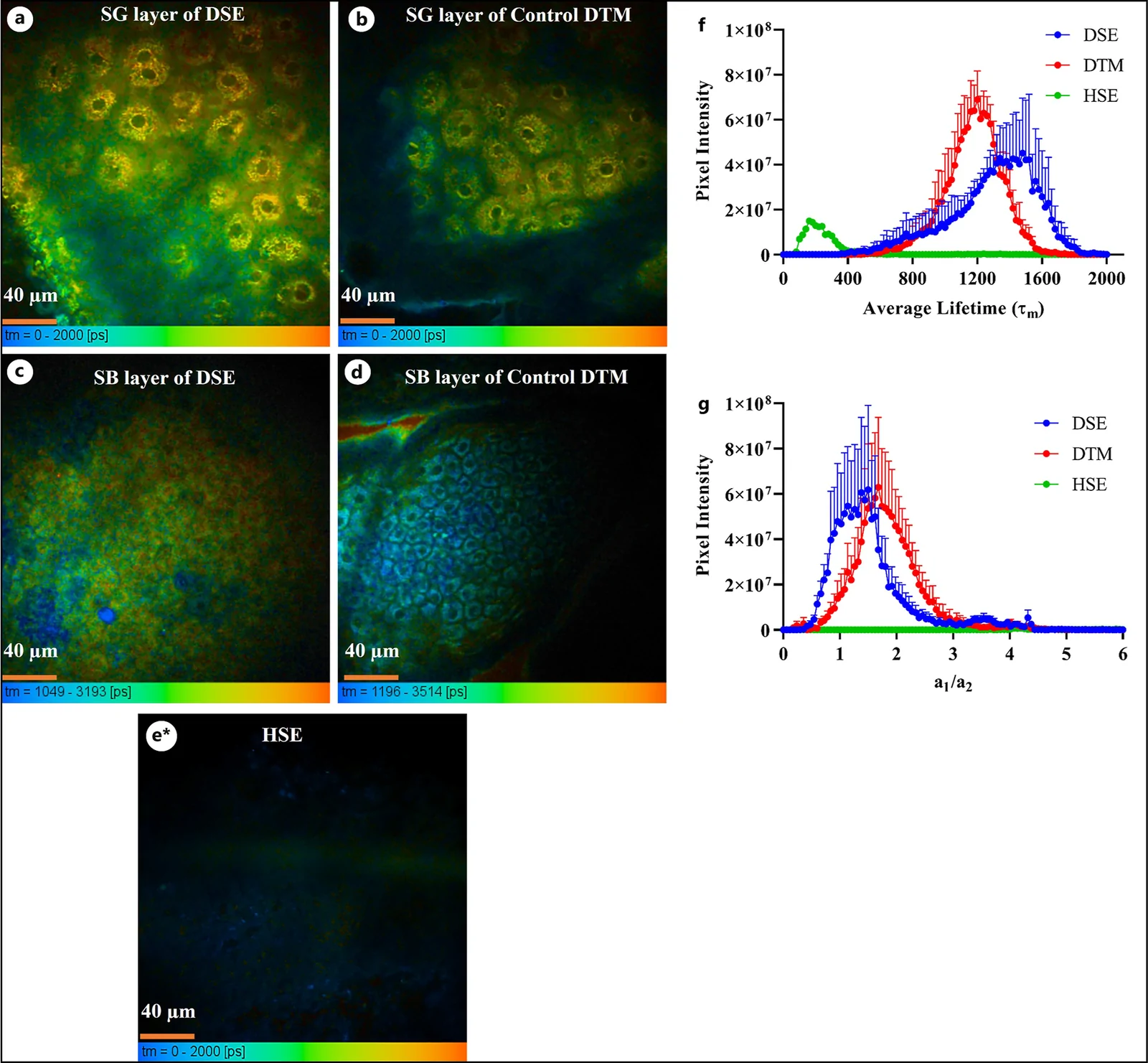
Representative pseudo-coloured FLIM images of stratum granulosum and stratum basale of DSE (**a**, **c**); of control DTM skin (**b**, **d**), respectively, imaged at an excitation wavelength of 740 nm. **e** HSE (*Specific structures cannot be identified in HSE). **f**, **g** Average pixel intensity versus fluorescence lifetime profiles of skin membranes.

**Table 1.** Average autofluorescence lifetime and a1/a2 ratio of NAD(P)H in 3 different donors

a1/a2 (mean±SD) (*n* = 3)			τm (mean±SD) (*n* = 3)		
control DTM skin	DSE	HSE (*n* = 1)	control DTM skin	DSE	HSE (*n* = 1)
2.0±0.2	2.2±0.4	6.3	1,173±37	1,229±74	246

#### Viability Testing by MTT Assay

Figure 3a shows that the dispase-separated epidermis (DSE) remained viable, as evidenced by its ability to convert MTT to insoluble formazan, comparable to the control DTM skin shown in Figure 3b. In contrast, the heat-separated epidermis (HSE) failed to metabolize MTT and remained unstained, indicating loss of metabolic activity (Fig. 3c). Quantitative analysis revealed that the percent viability of the DSE was lower than that of the control DTM skin (Fig. 3e). This difference may be attributed to the presence of viable dermal components in the control DTM skin, which can also contribute to MTT reduction and formazan formation, as illustrated in Figure 3d. Results in Figure 3e shows that viability remaining in Donor 2 is lower than other two donors which might be because of higher donor age which may have an impact of skin viability.

**Fig. 3.**
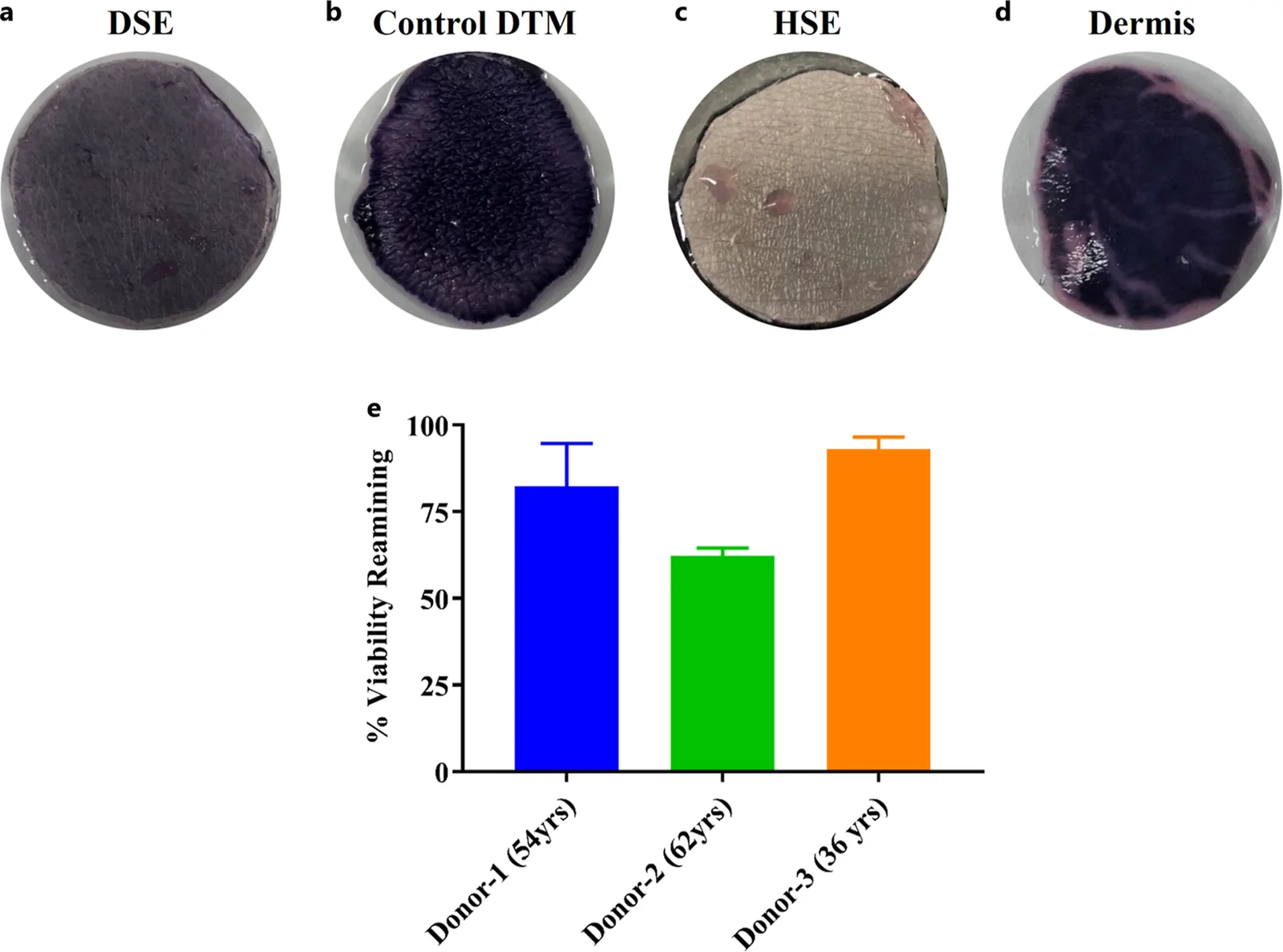
Representative images of after 90 min incubation in MTT solution. **a** Dispase separated skin. **b** Control DTM skin. **c** HSE. **d** Dermis. **e** Viability of DSE as a percentage of control DTM skin in three donors (mean ± SD *n* = 3) assuming 100% viability of control DTM skin.

#### H&E Staining

The 100× H&E-stained images in Figure 4 demonstrate that the dispase-separated epidermis (DSE) retains structural integrity and cellular morphology comparable to control DTM skin. The separation plane following dispase II treatment is clearly localized at the dermal–epidermal junction, with no evidence of cellular disruption in the viable epidermis. In contrast, heat-separated epidermis (HSE) exhibited visible loosening of the stratum corneum and stretched, disorganized epidermal layers (Fig. 4c), consistent with previously reported thermal effects on collagen softening below boiling temperature [45].

**Fig. 4.**
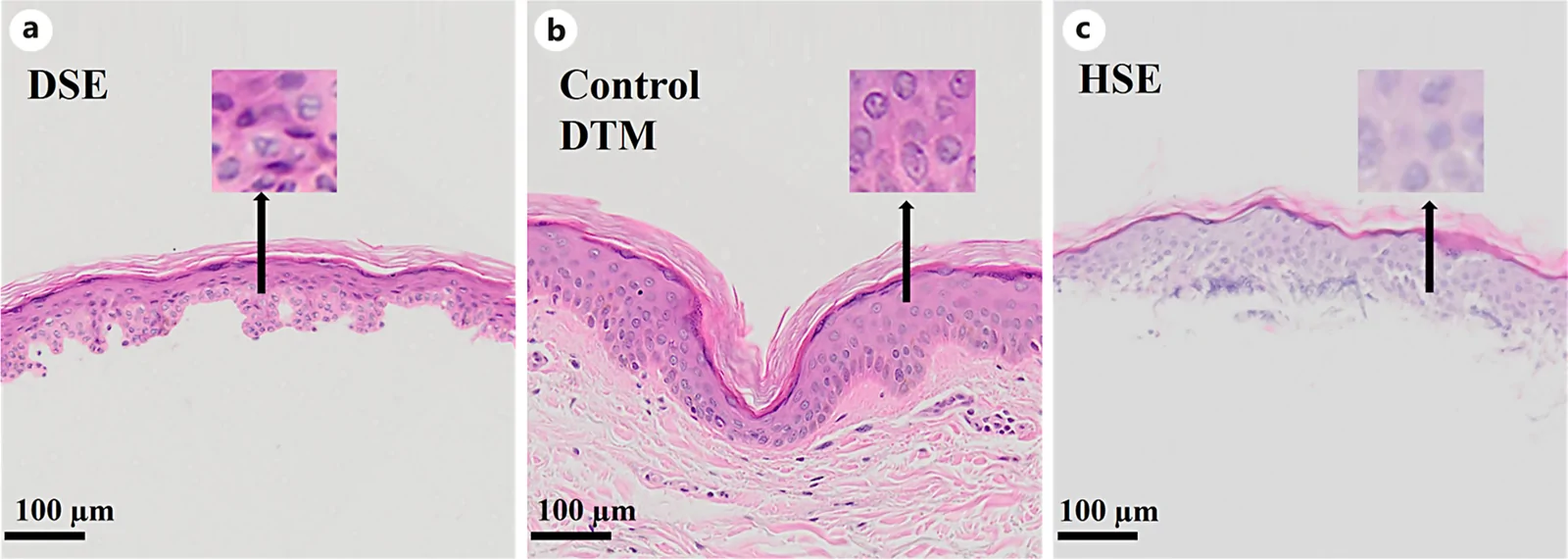
Hematoxylin and eosin staining of formalin fixed paraffin-embedded sections of DSE skin (**a**); control DTM (**b**); and HSE (**c**). Scale bar = 100 µm.

Hematoxylin, a basic dye, stains basophilic structures such as nucleic acids and nuclei blue to violet, while eosin, an acidic dye, stains acidophilic cytoplasmic proteins pink. As shown in Figures 4a and b, DSE and control DTM skin displayed normal hematoxylin and eosin staining patterns, with preserved nuclear and cytoplasmic compartments. In contrast, HSE (Fig. 4c) exhibited predominantly blue staining beneath the SC, indicating nuclear presence but a marked loss of cytoplasmic eosinophilia. This suggests degradation or loss of cytoplasmic content, further supporting compromised viability in the HSE.

### Mean Intensity in A Boxed Region over the Entire Z-STACK of RD123 Diffusion

Fluorescence intensity profiles from all 10 defined regions of interest (ROIs) across different epidermal depths – corresponding to the stratum basale (SB), stratum spinosum (SS), stratum granulosum (SG), and stratum corneum (SC) – were plotted over time for each donor (Fig. 5). In all three donors, the inhibitor-treated DSE showed faster diffusion and greater RD123 intensity in the upper SB layers compared to untreated control skin (Fig. 5a vs. b). Figure 5c shows the intensity of RD123 in HSE membrane for all three donors. Results show that was intensity was lower compared to DSE in all donors except in Donor 1.

**Fig. 5.**
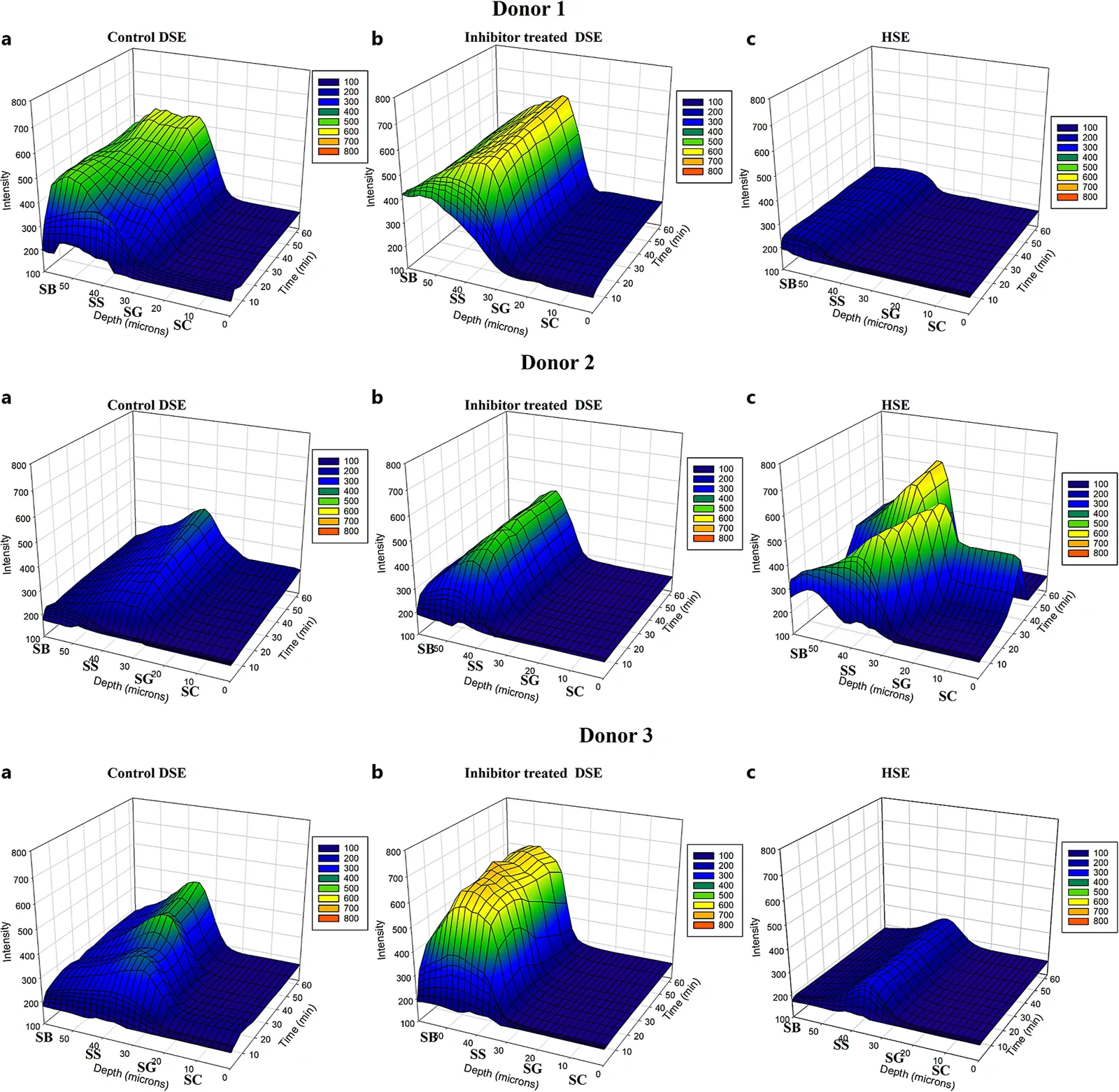
Intensity versus depth versus time 3D mesh plots of control DSE (**a**); inhibitor-treated DSE (**b**); HSE (**c**). Mean profiles from all 10 boxes combined for each treatment and for each donor.

Figure 6 further illustrates the averaged fluorescence intensities across three donors in different layers of the skin. At 8 min, the RD123 intensity was in the SG was higher in inhibitor-treated skin (434 arbitrary units) compared to control skin (320 arbitrary units) (Fig. 6a), indicating more rapid and extensive diffusion in the presence of verapamil. Results in Figure 6a also shows that intensity was higher in early time points in both SB and SS (shown in two representative SS layers in Fig. 6) layers of skin treated with inhibitor. At later time points (Fig. 6b), intensity levels in treated and control samples converged, suggesting the primary effect of P-gp inhibition is on early diffusion kinetics.

**Fig. 6.**
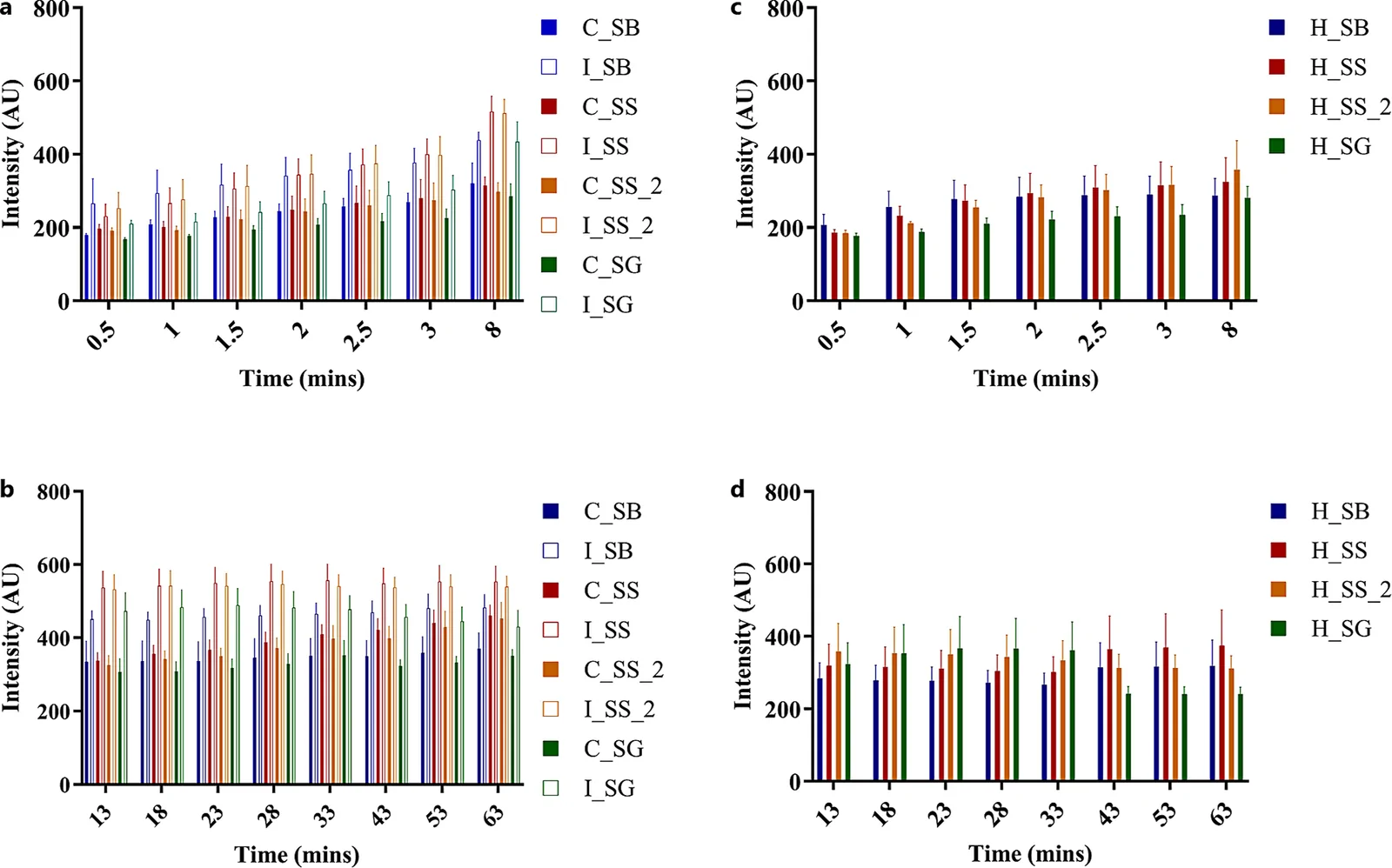
**a**, **b** Intensity versus time histogram plots of RD123 diffusion in control skin and inhibitor treated skin at different time points. **c**, **d** Intensity versus time at different time points in HSE. C in legend indicates control epidermis; I in legend indicates inhibitor treated epidermis; H in the legend indicates HSE (mean ± SEM *n* = 3 donors); 2 in legend indicates second representative layer of that specific layer.

Two-way ANOVA with Šídak’s multiple comparisons test confirmed that RD123 intensity was significantly higher in inhibitor-treated epidermis than in control epidermis in every viable epidermal layer examined: stratum basale (mean difference 107.7 a.u.; 95% CI 66.1–149.4; *p* < 0.0001), stratum spinosum (127.6 a.u.; 95% CI 85.9–169.3; *p* < 0.0001; and 136.1 a.u.; 95% CI 94.5–177.8; *p* < 0.0001 for the two representative SS layers), and stratum granulosum (106.8 a.u.; 95% CI 65.1–148.5; *p* < 0.0001). These results confirm that P-gp inhibition significantly increases RD123 accumulation across the viable epidermis (Table 2).

**Table 2.** Two-way ANOVA with Šídak’s multiple comparisons test of mean RD123 intensity between control and inhibitor-treated viable epidermis in each layer (*n* = 3 donors)

Šídák's multiple comparisons test	Mean diff.	95.00% CI of diff.	Below threshold?	Summary	Adjusted *p* value
C_SB vs. I_SB	−107.7	−149.4 to −66.07	Yes	****	<0.0001
C_SS vs. I_SS	−127.6	−169.3 to −85.91	Yes	****	<0.0001
C_SS_2 vs. I_SS_2	−136.1	−177.8 to −94.46	Yes	****	<0.0001
C_SG vs. I_SG	−106.8	−148.5 to −65.10	Yes	****	<0.0001

Negative mean differences indicate higher intensity in inhibitor-treated skin.

****p < 0.0001.

In contrast, HSE exhibited consistently lower RD123 intensities than viable membranes. At 60 min, the average SG intensity in HSE was 240 arbitrary units compared to 351 arbitrary units in control DSE (Fig. 6c–d), confirming reduced diffusion in non-viable membranes. These results provide qualitative evidence that diffusion dynamics differ significantly between viable and non-viable epidermis and further support the role of P-gp transporters in modulating early-phase diffusion of RD123 in viable skin layers following pharmacological inhibition.

#### Intensity of Viable Epidermis Layers at Various Time Points in Different Membranes

Figure 7 presents representative pseudo-coloured images of RD123 diffusion through different skin layers at selected time points, acquired from a single donor. Images from control skin (Fig. 7a), inhibitor-treated skin (Fig. 7b), and heat-separated epidermis (HSE; Fig. 7c) were captured using multiphoton microscopy, pseudo-coloured for fluorescence intensity, and

**Fig. 7.**
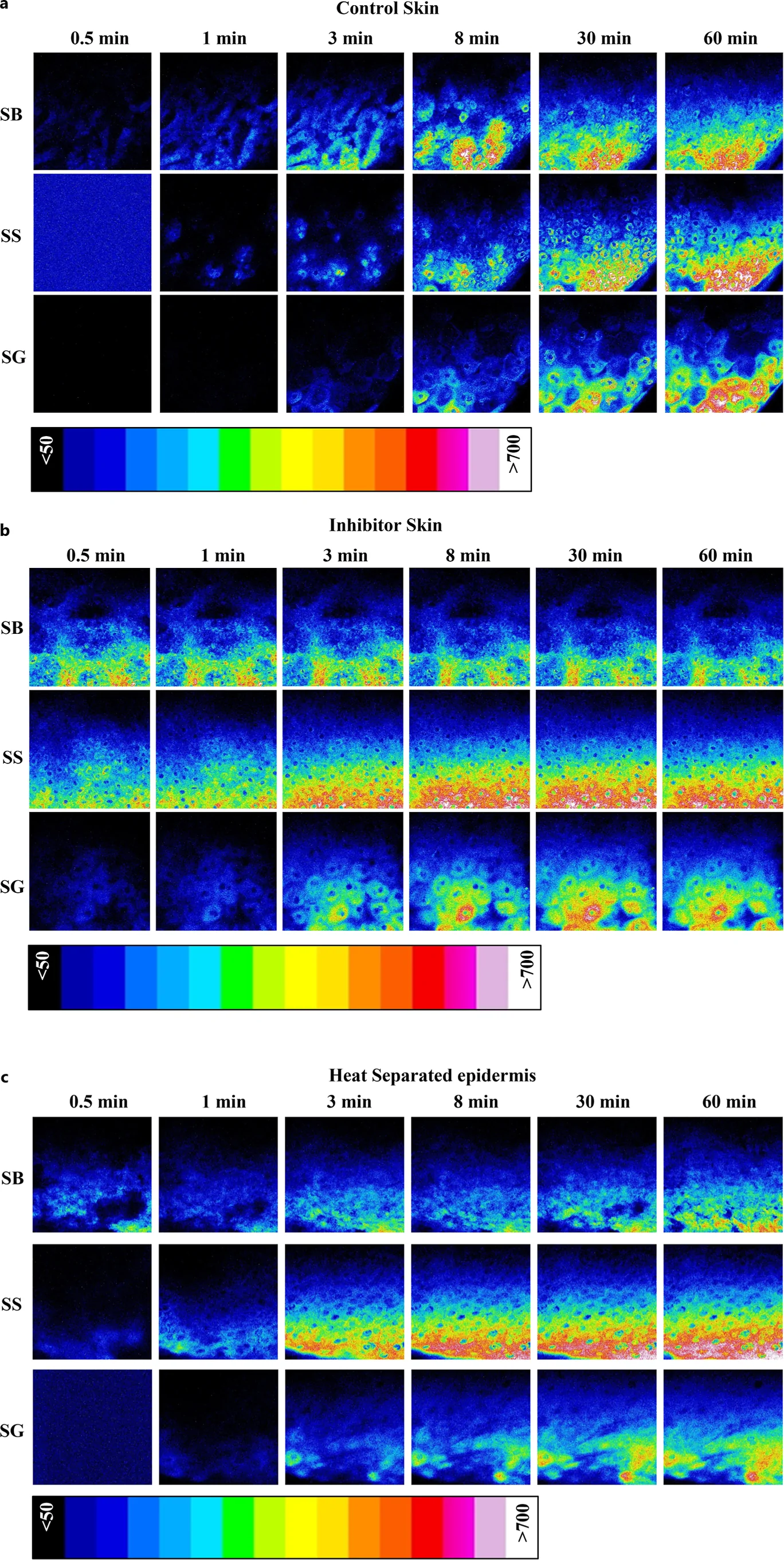
Representative images showing the change in RD123 intensity in the three layers of control DSE (**a**); inhibitor-treated DSE (**b**); HSE (**c**).

through viable layers. In contrast, inhibitor-treated skin displayed RD123 presence in all layers by 30 s, consistent with enhanced diffusion due to P-glycoprotein (P-gp) inhibition and reduced efflux in the viable epidermis.

In HSE samples, RD123 diffusion was detected in the SB and SS at 1 min and in the SG by 3 min. However, cellular morphology was notably disrupted, with loss of structural definition attributed to the thermal damage from the heat-separation process. This contrasts with the preserved architecture in the dispase-separated viable epidermis and further supports the importance of using physiologically intact models to study molecular transport in skin.

## Discussion

Quantifying molecular diffusion in the viable epidermis (VE) is essential for understanding local drug disposition yet remains technically challenging due to the difficulty of isolating viable, structurally intact membranes [10]. Traditional in vitro permeation uses (most often) full thickness or dermatomed skin while in vitro penetration often focuses on the SC by (most often) tape stripping, overlooking the pharmacokinetically relevant role of the VE. Furthermore, active transport mechanisms, including those mediated by P-glycoprotein (P-gp) and other ABC transporters, are increasingly recognized as modulators of drug retention and clearance within skin layers [31, 33]. In cutaneous drug transport, P-gp acts as an active barrier by extruding substrate compounds from keratinocytes, thereby limiting intracellular accumulation and restricting drug penetration into deeper skin layers and systemic circulation. This transporter functions in concert with the stratum corneum and cutaneous metabolic enzymes to regulate local and transdermal drug disposition. Variations in P-gp expressions or activity may, therefore, significantly influence the pharmacokinetics, efficacy, and safety of topical and transdermal drug delivery systems.

In systemic organs such as the liver, intestine, and kidney, trans-porter-mediated intrinsic clearance values are routinely measured and incorporated into physiologically based pharmacokinetic (PBPK) models to predict drug disposition [46]. A comparable approach in skin requires functional, quantitative data from viable epidermis. In this study, we demonstrate the application of a dispase-separated viable epidermal (DSE) membrane for assessing solute diffusion and transporter activity using a fluorescence-based imaging platform.

Our previous work validated the use of DSE membranes to study metabolic function of cutaneous esterases [47]. In the current study, we extended this model to assess active transport using rhodamine 123 (RD123), a known fluorescent substrate of P-gp [31, 34, 35]. Rhodamine 123 is a small molecule with a molecular weight of 344 Daltons and moderately lipophilic cationic compound (logP 1.06) [48] that can passively diffuse across biological membranes and partition into lipid-rich skin layers. In the viable epidermis, its distribution reflects a balance between passive diffusion driven by these physicochemical properties and active efflux via P-glycoprotein (P-gp), for which rhodamine 123 is a well-established substrate. Consequently, P-gp activity can significantly influence rhodamine 123 accumulation and should be considered when interpreting its use as a transport probe. Viability of the epidermal membrane was confirmed through multiphoton tomography-fluorescence lifetime imaging (MPT–FLIM), MTT assays, and histological staining, supporting its utility for mechanistic studies of cutaneous drug transport.

Using multiphoton microscopy (MPM), we visualized RD123 distribution across viable epidermal sublayers. Pretreatment with verapamil, a well-characterized P-gp inhibitor [36], resulted in a marked increase in fluorescence intensity and accelerated diffusion of RD123 compared to untreated controls. These findings suggest that inhibition of P-gp reduces RD123 efflux, thereby increasing intracellular accumulation and altering its rate of movement across the epidermal layers. Notably, diffusion from the suprabasal (SB) region toward the SC was enhanced, consistent with reduced trans-porter-mediated retention at the point of application. A limitation of the present study is that the experimental configuration does not replicate the physiological direction of topical drug penetration. For imaging and viability considerations, the viable epidermis was oriented such that solute diffusion occurred from the stratum basale toward the suprabasal layers, rather than from the stratum corneum toward deeper tissue as occurs in vivo. This inverted orientation was deliberately chosen to enable high-resolution live imaging of metabolically active keratinocytes and to preserve functional transporter activity within the viable epidermis. Importantly, once the stratum corneum barrier is bypassed, transport within the viable epidermis is dominated by diffusion through hydrated tissue and by cellular uptake and efflux processes. Under these conditions, diffusion kinetics are not expected to be inherently direction-dependent, if tissue architecture and transporter expression remain intact. Accordingly, the configuration used here is appropriate for mechanistic investigation of intra-epidermal transport, while acknowledging that it does not capture the full complexity of in vivo topical drug delivery across intact skin.

In contrast, heat-separated epidermis (HSE) showed markedly reduced RD123 intensity and lacked defined cellular architecture, confirming that membrane integrity and viability are critical for accurately assessing solute distribution. Structural disruption in HSE, likely due to denaturation during separation, prevents cellular uptake and likely alters membrane permeability, emphasizing the limitations of heat-based separation methods for transporter studies.

In skin specifically study by Sleeman et al. shows basal-layer keratinocytes efflux rhodamine 123 in a time-dependent, P-gp-dependent manner [49], and study by Giacone et al. in mice skin show pharmacological P-gp inhibition with elacridar increased accumulation of the substrate drug paclitaxel in the viable epidermis by ∼ 1.4-fold while reducing dermal and transdermal passage [50]. In this study, inhibition by verapamil an increase in stratum granulosum intensity from 320 to 434 a.u. at 8 min, ∼1.4-fold; significant across all viable-epidermis layers, *p* < 0.0001) is, therefore, of the same order as, though generally smaller than, effects reported in cell-line models. This is expected, given that P-gp (MDR1/ABCB1) is expressed only weakly-to-moderately in the epidermis and is considerably more abundant in dermal vasculature, sweat ducts and immortalized cell lines [26, 51]. Our results are further concordant with functional demonstrations of cutaneous P-gp using orthogonal approaches, including diffusion-cell and “skin-on-a-chip” verification of P-gp activity at the dermal barrier [28]and P-gp–dependent transdermal absorption of topical corticosteroids [29]. Unlike these cell-line methods and animal tissue studies, which report efflux ratios, IC50 values or Franz-cell fluxes, the imaging approach presented here resolves transporter modulated substrate distribution at the level of individual epidermal sublayers within an intact, viable membrane.

Although transcriptomic and proteomic data support the expression of ABC and solute carrier transporters in human skin [27, 52, 53], direct functional data remain limited. Mechanistic models such as MPML MechDermA (Certara) [54] and TCAT™ (GastroPlus) [55] provide frameworks for simulating cutaneous absorption, but are currently constrained by insufficient kinetic input and lack of measured transporter mediated in vitro*/*ex vivo intrinsic clearance for viable skin layers. Our findings provide novel, in situ evidence of active transporter function in intact viable epidermis and support the integration of such data into predictive dermal pharmacokinetic models.

Open flow microperfusion has been applied to assess dermal drug disposition in vivo [56], but lacks cellular resolution. In contrast, the imaging-based method presented here enables localization of solute distribution within defined epidermal sublayers, offering insights into both passive diffusion and trans-porter-mediated processes at the cellular level.

While the use of fluorescent probes such as RD123 limits current application to a subset of molecules, the approach can be extended by combining fluorescence microscopy with complementary techniques such as confocal Raman spectroscopy [57, 58] to quantify non-fluorescent drugs. Further development of time-resolved, high-resolution imaging at sub-minute intervals may enable detailed kinetic mapping of solute movement through intracellular and intercellular compartments. The values reported here are relative fluorescence intensities from a small donor set (*n* = 3) rather than absolute transport rates, and inter-donor variability in P-gp expression is expected. These constraints define the limits of the present comparison while supporting the qualitative conclusion that functional P-gp activity modulates early-phase RD123 distribution in the viable human epidermis.

This platform offers a practical and physiologically relevant tool to study drug diffusion in viable human epidermis and evaluate the impact of active transport. Future applications may include rapid assessment of permeability and retention for candidate compounds based on molecular weight or lipophilicity, and improved prediction of drug delivery profiles for both innovator and generic topical products. Importantly, the present findings have clear implications for in vivo drug delivery. The ability to quantify diffusion and trans-porter-mediated efflux within the viable epidermis provides a mechanistic basis for understanding local drug disposition following topical or transdermal administration in humans. Integration of the functional parameters derived from this platform into physiologically based pharmacokinetic (PBPK) models may improve prediction of dermal and systemic exposure. Ultimately, this approach may support the design and optimization of topical formulations and guide translation from ex vivo systems to in vivo performance.

## Conclusion

This study establishes a viable epidermal membrane model, generated via dispase II separation, as a robust platform for evaluating molecular diffusion and active transport in human skin. By integrating multiphoton microscopy with quantitative image analysis, we demonstrated P-glycoprotein (P-gp)-mediated modulation of rhodamine 123 distribution, confirming functional transporter activity in the viable epidermis under physiologically relevant conditions. These findings contribute to the growing body of evidence that cutaneous transporters influence local drug disposition, with important implications for both therapeutic efficacy and systemic safety of topically applied drugs. The methodology described here offers a translational tool to support early-phase screening, mechanistic studies, and the refinement of mathematical models for dermal absorption. From a regulatory perspective, incorporating functional data on transport and diffusion into model-based approaches may enhance bioequivalence assessments and inform formulation development for both innovator and generic dermatological products.

## Acknowledgments

We acknowledge the histology and microscopy core facilities of the Translational Research Institute for their support. We acknowledge Masood Ali for helping us to analyze MPT-FLIM data.

## Statement of Ethics

The study was conducted in accordance with the Declaration of Helsinki. The acquisition and use of the donated skin were approved by the Metro South Hospitals and the University of Queensland Human Research Ethics Committees (Approval No.: 2008001342). Written informed consent was obtained from all subjects involved in the study.

## Conflict of Interest Statement

The authors declare no conflict of interest.

## Funding Sources

This research received no external funding. Michael Roberts acknowledges fellowship support from the National Health and Medical Research Council of Australia (#1107356). This research was carried out at the Translational Research Institute, Woolloongabba, QLD 4102, Australia. The Translational Research Institute is supported by a grant from the Australian Government.

## Author Contributions

Conceptualization and investigation: K.C.T., J.E.G., Y.H.M., and M.S.R.; methodology: K.C.T., J.W, J.E.G., Y.H.M., and M.S.R.; script writing: J.W; formal analysis: J.W and K.C.T; writing – original draft preparation and visualization: K.C.T.; writing – review and editing: J.E.G., Y.H.M., and M.S.R.; supervision: Y.H.M. and M.S.R.; and funding acquisition: M.S.R. All authors have read and agreed to the published version of the manuscript.

## Data Availability

All data generated or analyzed during this study are included in this article and its supplementary material files. Further enquiries can be directed to the corresponding author.
